# S15-4 Challenges in translating research from controlled to real-life settings: preliminary results and evaluation of the “Take your brain for a walk” study

**DOI:** 10.1093/eurpub/ckad133.075

**Published:** 2023-09-11

**Authors:** Pauline Hotterbeex, Melanie Beeckman, Sebastien Chastin, Greet Cardon, Jannique van Uffelen

**Affiliations:** KU Leuven, Belgium; Ghent University, Belgium; Ghent University, Belgium; Glasgow Caledonian University, Scotland; Ghent University, Belgium; Ghent University, Belgium; KU Leuven, Belgium

## Abstract

**Purpose:**

Developing strategies to promote healthy cognitive ageing is a priority given the growing ageing population. Recent research has shown that cognitive activity (CA) combined with physical activity (PA) improves cognitive functioning. However, most studies on PA+CA have been conducted in controlled settings, and there is a need for real-life PA+CA interventions. We developed a real-life cognitively enriched walking program “Take your brain for a walk” in co-creation with older adults and experts. Currently, we are conducting a six-month randomized controlled trial (RCT), including an outcome and process evaluation.

**Methods:**

Participants are cognitively healthy community-dwelling adults aged 65+ years. They were randomly assigned to a: 1) cognitively enriched walking program (PA+CA) (performing cognitive tasks while walking); 2) walking program (PA only); or 3) passive control group. Both walking programs consist of two supervised 60-minute group-based walking sessions per week, with a total duration of six months (October 2022-April 2023). Memory and executive functioning are assessed using the Cambridge Neuropsychological Test Automated Battery at baseline (T0), three months (T1), six months (T2) and twelve months (T3; T1-T3 2023). Furthermore, a mixed-method process evaluation will be implemented based on the framework of Saunders et al. (2005), including a questionnaire and a semi-structured interview for the coaches and participants of the walking programs.

**Results:**

At baseline, 148 participants were included with a mean age of 70.4 (4.3) years, 70.9% was female. Seventeen participants dropped out in the first 3 months of the study. Three- and six-months effects (data from T0, T1 and T2) of the program on memory and executive functioning will be presented. We expect to see positive changes in cognitive functioning in both the PA+CA and PA arms, with larger effects for the PA+CA arm. Furthermore, the process evaluation results regarding fidelity, dose (delivered or received), satisfaction, adherence and context will be presented. The focus will be on the challenges of research in real-life settings.

**Conclusions:**

Findings will inform future lifestyle programs to promote cognitive functioning by simultaneously focusing on PA and CA for older adults in real-life settings.

**Support/Funding Source:**

This project is funded by the Research Foundation Flanders (FWO).

